# Matrix Metalloproteinases as Biomarkers and Treatment Targets in Mesothelioma: A Systematic Review

**DOI:** 10.3390/biom11091272

**Published:** 2021-08-25

**Authors:** Danijela Štrbac, Vita Dolžan

**Affiliations:** 1 Institute of Oncology, Zaloška 2, 1000 Ljubljana, Slovenia; dstrbac@onko-i.si; 2 Pharmacogenetics Laboratory, Institute of Biochemistry and Molecular Genetics, Faculty of Medicine, University of Ljubljana, Vrazov trg 2, 1000 Ljubljana, Slovenia

**Keywords:** mesothelioma, metalloproteinases, genetic variability, biomarker, drug target

## Abstract

Metalloproteinases (MMPs) have an important role in tissue remodeling and have been shown to have an effect on tumor progression, invasion, metastasis formation, and apoptosis in several tumors, including mesothelioma. Mesothelioma is a rare tumor arising from pleura and peritoneum and is frequently associated with asbestos exposure. We have performed a systematic search of PubMed.gov and ClinicalTrials.gov databases to retrieve and review three groups of studies: studies of MMPs expression in tumor tissue or body fluids in patients with mesothelioma, studies of MMPs genetic variability, and studies of MMPs as potential novel drug targets in mesothelioma. Several studies of MMPs in mesothelioma tissues reported a link between higher expression levels of commonly studied MMPs and clinical parameters, such as overall survival. Fewer studies have investigated genetic variability of *MMP* genes. Nevertheless, these studies suggested that certain genetic variants in *MMP* genes can have either protective or tumor-promoting effects on mesothelioma patients. MMPs have been also reported as novel drug targets, but so far no clinical trials of MMP inhibitors are registered in mesothelioma. In conclusion, MMPs play an important role in mesothelioma, but further studies are needed to elucidate the potentials of MMPs as biomarkers and drug targets in mesothelioma.

## 1. Introduction

Mesothelioma of pleura and peritoneum is a rare disease, linked to asbestos exposure in more than 80% of the cases. The incidence of mesothelioma is rising globally [1]. Considering that the peak exposure to asbestos was in the 1970s and 1980s, the long latency period that can last up to thirty years has produced the highest mesothelioma incidence in the last decade [2].

The diagnosis and treatment of mesothelioma is challenging. The treatment is based on a trimodal approach of surgery, chemotherapy, and radiation; however, the estimated median survival remains around 9–12 months [3]. Patients with epitheloid mesothelioma have a somewhat better overall survival over patients with biphasic and sarcomatoid subtypes. Newer therapies, such as immunotherapy, are emerging [4].

The increasing incidence of mesothelioma and the poor prognosis, despite the multimodal treatment calls for the identification of novel biomarkers that will allow earlier diagnosis, will have prognostic value and/or will predict the response to treatment. Mesothelioma biomarkers can be roughly divided into soluble glycoproteins (mesothelin, fibulin), genetic biomarkers (DNA markers, such as single nucleotide polymorphisms (SNPs), RNA expression levels, epigenetic biomarkers (DNA methylation, chromatin modifications, non-coding RNAs, such as microRNAs), and newly emerging proteomic biomarkers [5,6].

Matrix metalloproteinases (MMPs) are a group of tissue-remodeling enzymes that have an important biological role in healthy tissues and in disease. There are 23 currently known MMPs in humans. These enzymes have a similar structure that consists of a prodomain, a catalytic domain, a hinge region, and a hemopexin domain. They are either secreted from the cell or anchored to the plasma membrane. MMPs can be divided into six groups on the basis of their specific substrates. These are collagenases (MMP1, MMP8, and MMP13), which primarily degrade interstitial collagenes; gelatinases (MMP2 and MMP9, gelatinase A and B, respectively), which degrade gelatin, collagens, and laminin; stromelysins (MMP3, MMP10, MMP11), which degrade extracellular matrix componenets and activate proMMP forms; matrylisins or endometrase (MMP7 and MMP26) which process cell surface molecules, such as pro-α-defensin, Fas-ligand, pro-tumor necrosis factor (TNF)-α, and E-cadherin; type I transmembrane proteins (MMP14, MMP16, MMP24); and glycosylphosphatidylinositol (GPI) anchored proteins (MMP-17 and MMP-25), which are tissue-specific and have collagenotlytic activity. Lastly, there is a miscallenous group, such as macrophage elastase (MMP12), MMP19, and enamelysin (MMP 20). MMP12 is essential in macrophage migration, MMP19 is a T-cell-derived autoantigen and MMP20 is significant in tooth enamel formation. In the scope of this review, we have focused on the groups of MMPs that have a role in cancer progression, such as gelatinases and trans membrane MMPs (TM-MMPs) [7,8]

While the primary function of MMPs in cancer is tissue remodeling and extracellular matrix turnover, they are involved in other mechanisms as well. MMPs in cancer promote tumor angiogenesis, especially MMP9, which regulates the release and activation of vascular endothelial growth factor (VEGF). Furthermore, they activate inflammatory mediators, such as tumor necrosis factor α (TNFα). Lastly, they are important regulators of tumor microenvironment and contribute to metastatic invasion of tumor cells via extracellular matrix remodeling and activation of VEGF release and tumor angiogenesis [9].

The most frequently investigated MMPs in mesothelioma are MMP2 (gelatinase A), MMP9 (gelatinase B), and MMP14. MMP2 is a zinc-dependent enzymatic complex with three fibronectin type II repeats in a catalytic site that allows binding of denatured type IV and V collagen and elastin. This enzyme can be activated extracellularly by proteases, or intracellularly by S-glutathiolation, which does not require proteolytical removal of the pro-domain. MMP2 proenzyme can be bound by the tissue inhibitor of metalloproteinase 2 (TIMP2), thus forming an inactive MMP2-TIMP2 complex.

The MMP9 proenzyme consists of five domains: a signal peptide, a propeptide, a catalytic domain with inserted three repeats of fibronectin type II domain, followed by a C-terminal hemopexin-like domain. Activation of MMP9 is primed by MMP3; however, MMP9 can also be activated autocatalytically [10,11].

MMP14 is a member of membrane-type MMPs (MT-MMPs) which contains a transmembrane domain and is therefore expressed on the cell surface rather than secreted. MMP14 is involved in activation of the proMMP2 and also plays a role in activation of mechanisms of tissue matrix remodeling, tumor angiogenesis, cell signaling, migration, and inflammation [12].

Expression of different MMPs was studied in mesothelioma tumor tissue, pleural effusions, or plasma samples. Other studies focused on genetic variability of the MMPs and investigated single nucleotide polymorphisms (SNPs) as potential diagnostic and prognostic markers in mesothelioma. MMPs are also becoming more and more interesting as potential treatment targets, and the evolving field of MMPs inhibitors is focusing on their anti-tumor potential.

**Figure 1:**
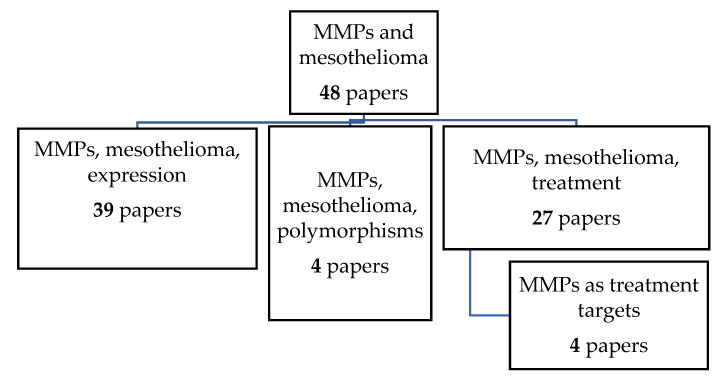
Stratification of papers considered in this review.

This review systematically summarizes findings on the role of MMPs as potential biomarkers and treatment targets in mesothelioma. We searched the available literature in the PubMed.gov [13] and ClinicalTrials.gov [14] databases. The PubMed.gov search was limited to the data published from the year 2000 up to the year 2021. We used the combination of keywords “matrix metalloproteinase and mesothelioma” or the combination of “MMP and expression”, “MMP and mesothelioma and biomarker”, “MMP and mesothelioma and polymorphisms”, and “MMP and mesothelioma and treatment”. The ClinicalTrials.gov database search was limited to the completed studies with the keywords “mesothelioma and metalloproteinase” and “cancer and metalloproteinase”. With regards to the biomarker studies, we have considered only studies that were performed on human samples or cell lines. Studies on mesothelioma animal models were included in the review of studies of MMPs inhibitors. We retrieved 48 papers exploring the role of MMPs in mesothelioma. We stratified these studies into three groups: (1) MMPs’ expression in tumor tissue or body fluids in mesothelioma; (2) MMPs’ genetic variability in mesothelioma; and (3) MMP’s as potential novel mesothelioma treatment targets (Figure 1).

## 2. MMPs Expression in Tissue and Body Fluids as Biomarker in Mesothelioma

The most important studies that investigated MMP expression in mesothelioma tissue and body fluids are summarized in Table 1.

Amati et al. investigated several blood biomarkers, including MMP2 and MMP9, with the goal of finding a potential biomarker for early detection of mesothelioma. Their study included 22 patients with mesothelioma, 94 high-risk asbestos-exposed subjects, and 54 healthy subjects. The expression levels of MMP2 and MMP9 were not significantly different between these groups of subjects. The best sensitivity and specificity for distinguishing the individual groups was achieved by the combination of other soluble biomarkers, such as 8-Hydroxy-2′-deoxyguanosine (8OHdG), vascular endothelial growth factor (VEGF), and soluble mesothelin-related peptides (SMRPs), indicating that they could have a better diagnostic value in mesothelioma [15,16]. However, there is a clinical application to MMPs in mesothelioma, since increasing MMP2 activity suggests poorer survival and is correlated with clinical parameters of diseases progression, such as weight loss [17].

Some other studies investigating MMPs in mesothelioma focused on the basic roles of MMPs in tumor invasion and progression [18,19]. Doi et al. investigated the expression of MT1-MMP and the invasion ability of the mesothelioma cell line established from a clinical specimen of a patient with mesothelioma. They have silenced the expression of MT1-MMP by RNA interference (RNAi) to assess the role of MT1-MMP in the invasive activity of mesothelioma cells. The invasion assay revealed that a high expression of MT1-MMP in mesothelioma cells was associated with more aggressive invasive activity. Besides confirming the role of MT1-MMP in the invasiveness of mesothelioma cells, this study also suggested that MT1-MMP could be a suitable molecular target for the suppression of the invasiveness of mesothelioma cells [20]. The role of MT1-MMP as a potential biomarker needs to be addressed in further studies.

Servais et el. investigated the correlation between mesothelin (MSLN) and MMP9 expression levels in tissue microarrays from patients with epithelioid mesothelioma. They observed that MSLN overexpression correlated with higher MMP-9 expression at individual core level. They concluded that there is a biological role for MSLN as a factor-promoting tumor invasion and MMP-9 expression in MSLN-expressing mesothelioma tissue [21].

Edwards et al. compared MMP2 and MMP9 expression levels and activity in homogenates of snap frozen samples of mesothelioma tissue (*n* = 35), inflamed pleura (IP, *n* = 12 s), and uninflamed pleura (UP, *n* = 1). Despite the low number of samples, they have observed a correlation between MMP levels and clinicopathological factors, and also with survival. Mesothelioma pro-MMP2 and active MMP2 levels were significantly higher than MMP9 levels. Active MMP2 levels were significantly higher in mesothelioma than in uninflamed pleura. MMP2 activity was similar in inflamed pleura and mesothelioma, but MMP9 activity was higher in inflamed pleura. Higher total and pro-MMP2 activity tended to be associated with poor survival; however, in combination with weight loss, they both reached significance as independent poor prognostic factors. MMP9 activities did not reach prognostic value [17]. This study clearly indicated that MMP2 is the most abundant gelatinase in mesothelioma, so it may play an important role in tumor growth and metastasis. Therefore, agents that could reduce its synthesis and/or activity could play a role in the management of mesothelioma.

The quest for better understanding of regulatory mechanism that control MMPs expression is the aim of the most recent study by Sakai et al. *MMP2* gene expression is regulated by DNA and histone methylation around the transcription start site and polycomb repressive complex 1 (PRC1) was shown to play and important role in the regulation of transcription via epigenetic mechanisms. Chromobox 6 (CBX6) is a subunit of PRC1 that mediates epigenetic gene repression and acts as an oncogene or tumor suppressor in a cancer type-dependent manner [22]. Transcriptome analysis suggested that CBX6 regulates sets of genes involved in mesothelioma migration and metastasis. Knockdown of CBX6 promoted MMP2 expression and invasion of mesothelioma cells. In human tissues, CBX6 was localized in the nuclei of normal mesothelium and benign mesothelioma; however, in malignant mesothelioma, the nuclear staining of CBX6 was lost. CBX6 was shown to be constantly unstable due to ubiquitination and protein degradation in invasive, but not non-invasive cells. These data suggest that proteasomal degradation of CBX6 may be related to mesothelioma progression [1].

The roles of MMPs expression and mesothelioma progression and invasion are becoming better understood and the correlation between MMPs expression and some clinical parameters, such as weight loss, have been established. However, recent studies have focused on elucidating genetic mechanisms that control MMPs expression in mesothelioma as these genetic mechanisms may be potential future targets for therapies.

**Table 1:** Metalloproteinase (MMP) expression in mesothelioma tissues or body fluids.

MMP	Biomaterial/Biosample	Sample Size	Endpoint	Major Findings	Reference
MMP2, MMP9	plasma	76	To evaluate different plasma peptides as biomarkers for early detection of mesothelioma	MMP2 and MMP9 had no diagnostic value	Amati et al. [15]
MMP2, MMP9	plasma	170	To evaluate MMP2 and MMP9 as soluble diagnostic markers	MMP2 and MMP9 had no diagnostic value	Amati et al. [16]
MMP14	tumor derived cell line	1	To evaluate the role of MT1-MMP in invasiveness of mesothelioma	Higher expression of MT1-MMP suggested higher local invasiveness of mesothelioma	Doi et al. [20]
MMP9	tissue microarray	211	To assess the influence of mesothelin on MMP9 expression in murine and human mesothelioma tissue	Overexpression of mesothelin influenced MMP9 levels in human mesothelioma	Servais et al. [21]
MMP2, MMP9	homogenised supernatants of snap frozen pleura	61	To determine the pro-MMP2, MMP2 and MMP9 prognostic potential in mesothelioma	Increased pro-MMP2 levels and MMP2 activity were associated with poor prognostic factor in conjugation with weight loss; MMP9 had no prognostic significance	Edwards et al. [17]
MMP2	mesothelioma cell line	1	To determine the role of CBX6 in expression of MMP2	Increased CBX6 degradation in mesothelioma cells promoted expression of MMP2	Sakai et al. [18]

## 3. *MMPs* Genetic Variability in Mesothelioma

The last decade in the research of MMPs in mesothelioma has focused on the genetic mechanisms controlling either the expression of MMPs or SNPs present in *MMPs* genes. The studies that investigated genetics of *MMPs* in mesothelioma are summarized in Table 2.

Crispi et al. have performed transcriptome analysis on nine samples of human pleural mesotheliomas and four normal pleura samples using Affymetrix microarrays containing probes for 39,000 human transcripts. The differentially expressed genes included several genes previously described as prognostic classifiers as well as novel genes that could play a role in tumor progression, including *MMP14*. The potential role of selected differentially expressed genes as biomarkers was further investigated in 70 mesothelioma patients, among which 45 presented with epithelioid, 11 with sarcomatoid, and 14 with mixed mesotheliomas. Expression of MMP14 was found to be the only parameter associated with overall survival. The calculated relative risk of death in mesothelioma patients with low MMP14 expression was significantly lower than in patients with high MMP14 expression [23].

As MMP2, MT1-MMP (MMP14), and tissue inhibitor of MMP2 (TIMP2) were associated with aggressive tumor progression and low survival rates in several tumor types, Zhong et al. investigated their gene expression, protein activation inhibition, and regulation via signaling pathways in six human mesothelioma and one mesothelial cell line. They have shown that mesothelial cells expressed MT1-MMP and that migration, but not proliferation, of mesothelioma cells depended on its presence and activity. They have shown that MT1-MMP is needed to activate pro-MMP2 to facilitate migration through extracellular matrix. They have also shown that p38 mitogen-activated protein kinase (MAP kinase) is involved in regulation of pro-MMP2 expression and that its phosphorylation is deregulated in mesothelioma cells [24].

Although most studies investigated MMPs expression levels as biomarkers in mesothelioma, few studies have focused on genetic variability as a source of interindividual variability of MMP expression and activity levels, and have investigated common functional SNPs in *MMPs* genes as potential biomarkers in mesothelioma.

Strbac et al. explored SNPs in *MMP2*, *MMP9*, and *MMP14* as baseline risk predictors in mesothelioma. In a study that included 236 mesothelioma patients and 161 healthy blood donors, carriers of at least one polymorphic *MMP2* rs243865 allele had significantly lower risk for mesothelioma. This association was even more pronounced in patients exposed to asbestos, indicating that this SNP could be used as a potential baseline risk predictor in mesothelioma [25].

In another study, Strbac et al. investigated these SNPs as prognostic biomarkers in a study that included 199 mesothelioma patients. The study showed association of *MMP9* rs2250889 with shorter time to progression and overall survival, while *MMP9* rs205449 had the opposite effect and was associated with longer time to progression [26]. These SNPs are of interest as potential prognostic biomarkers in mesothelioma; however, their biological role needs further elucidation as they were not associated with serum MMP9 levels in a later study. Interestingly, among the investigated *MMP9* SNPs, only rs17576 showed association with serum MMP9 levels before treatment. Median serum MMP9 levels differed significantly before and after treatment of MM, but failed to reach significance as a standalone biomarker of treatment response in mesothelioma [2]. MMP9 serum levels and *MMP9* polymorphisms should be further tested as a composite non-invasive diagnostic and prognostic biomarker in mesothelioma.

**Table 2:** Metalloproteinase (MMP) genetic variants in mesothelioma tissues or body fluids.

MMP	Biomaterial/Biosample	Sample Size	Endpoints	Major findings	Reference
MMP14	pleural tissue	13	To find a potential biomarker among 39,000 mesothelioma transcripts	Relative risk of death was lower in mesothelioma patients with lower MMP14 expression	Crispi et al. [23]
MMP2 MMP14	cell line	1	To elucidate if TGFβ1 and p38 influence MMP2 expression in mesothelioma cell lines	Phosphorylation of p38 kinase up- regulated the expression of MMP2 in mesothelioma cell lines	Zhong et al. [24]
MMP2 MMP9 MMP14	germline DNA	199	To investigate *MMP2, MMP9* and *MMP14* polymorphisms as potential prognostic biomarkers	Certain polymorphisms within the *MMP9* gene were associated with longer time to progession and survival, while others had the opposite effects	Strbac et al. [25]
MMP2 MMP9 MMP14	germline DNA	397	To find if *MMP2, MMP9* and *MMP14* polymorphisms influence risk for mesothelioma	MMP2 rs243865 had a protective role in pleural mesothelioma development	Strbac et al. [26]
MMP9	germline DNA and serum samples	110	To find if *MMP9* polymorphisms correlate with MMP9 serum levels	rs17576 correlated with MMP9 serum levels before mesothelioma treatment	Strbac at al. [27]

## 4. MMPs as Potential Novel Mesothelioma Treatment Targets

Although the prospects of immunotherapy have finally reached mesothelioma, there is still a great need for novel therapeutic approaches. Immunotherapy is an integral part of cancer treatment in many more common cancer types, such as lung cancer and melanoma; however, in mesothelioma, the improvements in outcomes are not as significant [28]. Therefore, MMPs became of huge interest as possible novel targets for treatment with MMP inhibitors.

Our literature and database search has, however, revealed that the studies of MMP inhibitors in mesothelioma are limited to human mesothelioma tissue lines and animal tumor models. We have found no completed or ongoing clinical trials related to MMPs in mesothelioma, although clinical trials of MMPs inhibitors are ongoing in other cancers [14]. There have been three clinical trials in lung cancer with MMP inhibitors. One of the trials used prinomastat as a synthetic MMP inhibitor in conjunction with the standard gemcitabine–cisplatin chemotherapy. Another trial used marimastat as a maintenance therapy after first line treatment. In the third lung cancer trial, an anti-MMP drug, S-3304, was used in conjunction with radiation therapy and paclitaxel–carboplatin doublet. However, these trials did not achieve clinical use in lung cancer. It could be debated that this group of potential therapeutic targets is under-investigated in the light of other (e.g., immunotherapy) treatment approaches [14]. This is evident especially in mesothelioma, which is a rare tumor. However, due to its poor prognosis and limited efficacy of current treatments, more efforts should be invested into research and trials of MMPs inhibitors in mesothelioma.

Currently investigated MMP inhibitors may be stratified into two main groups: synthetic and natural inhibitors. Different classes of synthetic inhibitors are investigated in clinical trials on humans; among them are synthetic peptides, non-peptide molecules, chemically modified tetracyclines, and bisphosphonates. The most investigated natural MMP inhibitors so far are isoflavonoids and shark cartilage [29].

The studies that investigated MMPs as potential drug targets in mesothelioma are summarized in Table 3.

Roomi et al. investigated a mixture of lysine, proline, ascorbic acid, and green tea extract as possible mesothelioma cell line inhibitors. Different doses were measured at which the mixture was effective. In a dose of 100 microgram per ml of mixture, 36% of MMP2 and MMP9 molecules were blocked and the mixture was not toxic to the mesothelioma cell line. The studied mixture also significantly inhibited MMP secretion and invasion of mesothelioma cell [30]. However, the rationale for choosing this mixture were not clear and its effects on metabolic and pharmacological pathways were not studied in detail.

Other studies focused on specific mechanisms of MMP inhibition. Ciaramella at al. investigated kisseptin (KiSS1), a metastasis suppressor that activates the G-protein coupled receptor (GPR54) and decreases the cells metastatic potential. Treatment with the KiSS1 peptide or with a synthesis peptide with longer half-life, i.e., the FTM080, significantly inhibited cell proliferation, migration, and invasion of mesothelioma cell lines. The same treatment also reduced the activity of MMP2 and MMP9, consequently leading to a marked reduction in the invasiveness of primary tumors and metastases [31].

Buommino et al. focused on 3-*O*-methylfunicone (OMF), a secondary metabolite produced by Penicillium pinophilum, which affects cell proliferation and motility in a variety of human solid tumors. Their study showed that OMF inhibited the motility of the mesothelioma cell line by modulating extracellular signal-regulated kinase (ERK) signalling activity, and affected alphaVbeta5 integrin and MMP-2 expression by inducing marked downregulation at both mRNA and protein levels [32].

In addition to novel inhibitory peptides, repurposed drugs could be used as potential MMP inhibitors in mesothelioma and other solid cancer. Bisphosphonates, in particular zoledronic acid, appear to directly inhibit the activity of several MMPs, including MMP9 [33]. Although bisphosphonates have proven to be an effective and safe treatment, especially in osteoporotic disease; their anti-proliferative effects were mainly investigated in vitro in cancer cell lines. A study in a mouse model showed that bisphosphonates also inhibit mesothelioma tumor growth and prolong the survival of mesothelioma-bearing mice [34]. These results support further studies of bisphosphonates as MMP inhibitors in mesothelioma, in particular as they were shown to accumulate in mesothelioma. Furthermore, in mouse mesothelioma cell lines, risedronate and zoledronate induced phosphorylation of p38 MAP kinase; this signaling pathway was shown to regulate MMP2 expression [35].

It has to be noted that all of the above cited studied investigating MMP inhibitors were performed on mesothelioma cell lines or mouse models of mesothelioma. However, the transition from a preclinical setting to clinical testing could be a promising one, since many of these drugs, such as bisphosphonates, are already available for other diseases.

## 5. Conclusions and Future Perspectives

MMP expression and genetic variation may have an important role in mesothelioma as prognostic biomarkers and as potential treatment target. The expression of MMPs in tumor tissue and body fluids of mesothelioma patients was explored in the past decade mainly as potential diagnostic and prognostic biomarkers. Studies of genetic pathways and their variability are also gaining interest not only as a potential source of genetic biomarkers, but also for enabling better understanding of genetic and epigenetic mechanisms that regulate MMPs expression. The future thus seems to be more complex and focused on novel treatment approaches. Modulators and regulators of MMP expression in different tissues seem to be the new frontier of studies aiming to find the novel drug targets and MMP inhibitors that could be used in the management of mesothelioma as well as in other solid cancers. After preparing this review, we conclude that there are numerous studies regarding MMPs and their genetic variables as prognostic biomarkers in other more common cancers, such as lung and colon cancer [36,37,38,39,40]. However, MMP inhibitors as potential therapeutic targets are under-investigated, even in these more common malignancies.

While mesothelioma is a rare disease in comparison to lung cancer, the death burden of this disease is still high and is increasing in middle- and lower-income countries [41]. We may, therefore, conclude that the need for new therapeutic approaches in mesothelioma is great, and that MMPs may be interesting, not only as biomarkers, but also as treatment targets.

## Figures and Tables

**Table 3:** Metalloproteinases (MMPs) as potential drug targets.

MMP	Biomaterial/Biosample	Endpoints	Major Findings	Reference
MMP2 MMP9 MMP12 MMP13	review	To review specific MMP inhibitors	Specific MMPs inhibitors can be repurposed as anticancer agents	Jablonska et al. [29]
MMP2 MMP9	MM cell line	To explore the effect of lysine, proline, ascorbic acid, and green tea extract mixture on MM cell growth	The mixture inhibited MMP secretion and MM cell growth	Waheed at al. [30]
MMP2 MMP9	MM cell line	To explore the effect of KiSS1 peptide on MMP2 and MMP9 activity	KiSS1 reduced MMP2 and MMP9 activity and had antiproliferative effect on mesothelioma cell line	Ciaramella et al. [31]
MMP2	MM cell line	To explore the role of 3-O-methylfunicone on MMP2 expression and cell mobility	The compound inhibited mobility of mesothelioma cells via multiple mechanism and MMP2 down regulation	Buommino et al. [32]
